# Evolution of nitrogen cycling in regrowing Amazonian rainforest

**DOI:** 10.1038/s41598-019-43963-4

**Published:** 2019-06-12

**Authors:** Viviane Figueiredo, Alex Enrich-Prast, Tobias Rütting

**Affiliations:** 1 Department of Botany, University Federal of Rio de Janeiro, 21941-971 Avenida Carlos Chagas Filho, Rio de Janeiro, Brazil; 2 Postgraduate Program in Geochemistry, University Federal Fluminense, 24020-007 Outeiro de São João Batista, Niterói, Brazil; 3 Postgraduate Program in Biotechnology, University Federal of Rio de Janeiro, 21941-971 Avenida Carlos Chagas Filho, Rio de Janeiro, Brazil; 4 Department of Environmental Change, Linköping University, 58183 Linköping, Sweden; 5 Department of Earth Sciences, University of Gothenburg, 405 30 Gothenburg, Sweden

**Keywords:** Element cycles, Ecosystem ecology, Geochemistry

## Abstract

Extensive regions of tropical forests are subjected to high rates of deforestation and forest regrowth and both are strongly affect soil nutrient cycling. Nitrogen (N) dynamics changes during forest regrowth and the recovery of forests and functioning similar to pristine conditions depends on sufficient N availability. We show that, in a chronosequence of Amazonian forests, gross nitrification and, as a result, nitrate-to-ammonium (NO_3_^−^: NH_4_^+^) ratio were lower in all stages of regrowing forests (10 to 40 years) compared to pristine forest. This indicates the evolution of a more conservative and closed N cycle with reduced risk for N leaking out of the ecosystem in regrowing forests. Furthermore, our results indicate that mineralization and nitrification are decoupled in young regrowing forests (10 years), such as that high gross mineralization is accompanied by low gross nitrification, demonstrating a closed N cycle that at the same time maintains N supply for forest regrowth. We conclude that the status of gross nitrification in disturbed soil is a key process to understand the mechanisms of and time needed for tropical forest recovery.

## Introduction

In the Brazilian Amazon region, almost 800 000 km^2^ of land has been deforested, mainly for soya bean cultivation, logging and cattle ranching^1^. The high rate of tropical deforestation led to global concern since these areas are a hot spot of biodiversity and have direct influence on the global climate through hydrology and exchange of greenhouse gases^2–5^. However, a large area of approximately 167 000 km^2^ previously deforested land has been abandoned after exploitation^6^ and secondary forests have established on that land^7^. The regrowth area in the Amazon is increasing^6^, but our current knowledge about nutrient availability, biogeochemical processes, and how the post-disturbance regeneration influences these processes is poorly understood^8^. Likewise, nutrient shortage in deforested areas is expected^9^, but the influence and magnitude of limitation, which can drive the recovery trajectory, on regrowth forest are still uncertain^10^.

Early secondary forests have high growth rates with rapidly increasing forest biomass^11^, even when N is apparently limited^12^. This indicates that feedback mechanisms on soil N availability exist, providing sufficient plant available N to maintain forest regrowth. Microbial processes, such as mineralization and nitrification, drive the soil N cycle and thereby control the amount of organic and inorganic N forms in soil^13,14^. Mineralization of soil organic matter (SOM) is responsible for inorganic N production in terrestrial ecosystems, which is important for plant N uptake that occurs mainly in inorganic form. The NH_4_^+^ released by mineralization also supports nitrification^15^, the oxidation of NH_4_^+^ to NO_3_^−^. These two inorganic N forms may have different fates in soils, as immobilization in biomass, leaching and gas losses^16^, and the occurrence and magnitude of these pathways might influence the forest growth^17^.

Davidson *et al*.^8^ investigated the N cycling recovery in secondary forest age chronosequences after agricultural abandonment in the Amazon region. These authors found indications for a conservative N cycling in soils of young successional tropical forests based on N and phosphorus (P) contents in leaves, litterfall and soils, low NO_3_^−^: NH_4_^+^ ratios as well as low nitrous oxide (N_2_O) emissions. However, the mechanistic changes in the soil N cycle during forest regrowth have not been studied in the Amazon Region. The actual dynamic of labile N in soils is best represented by gross soil N cycle dynamics, such as gross N mineralization and nitrification, since the gross transformations directly control the inorganic N availability for plants growth. Therefore, quantifying the gross N transformations in tropical regrowth forest soils is an important step in managing and enhancing abandoned managed areas, which also provides valuable information for model implementation.

We evaluated the gross soil N cycling in four forests, including one pristine forest and one regrowth forest (40 years old) located inside and two regrowth forests (10 and 20 years old) near the Ecological Station of Cuniã in the state of Rondônia, Western Amazonia, with focus on gross N mineralization and gross nitrification. The slash-and-burn practice was applied in all three regrowth areas studied here. The ^15^N pool dilution technique using the “virtual soil core” approach^18^ was used to quantify *in situ* gross N processes rates. Predominant soil type of the investigated forests is Plinthosol^19^, soil texture in the pristine forest is sandy loam with 55.4% (±4.4) sand, 39.1% (±4.8) silt and 4.9% (±0.7) clay (mean ± SD; N = 7). The vegetation is dominated by hardwood with abundance of palms^20^.

## Results and Discussion

### Sustained production of plant available N in tropical regrowth forests

**Figure 1:**
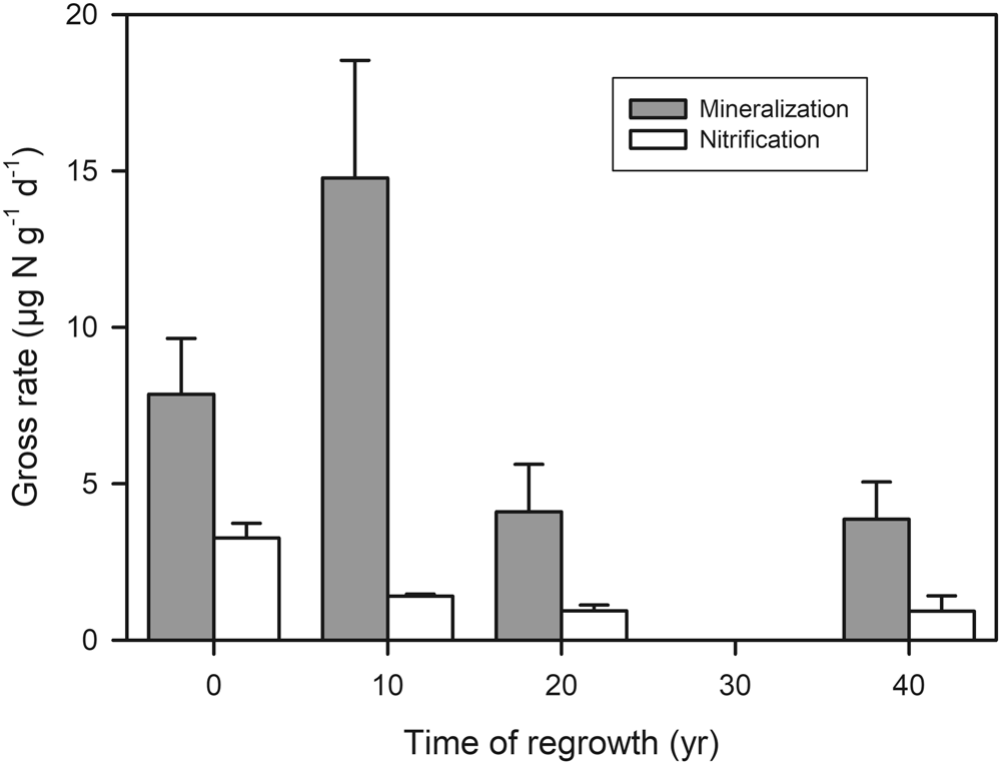
Gross N mineralization (gray bars) and nitrification (white bars) rates (µg N g^−1^ SDW d^−1^; Mean ± SE) in four forest soils at Cuniã Ecological Station, Rondônia, with one pristine forest (set to t = 0 years) and three regrowth forests (10, 20 and 40 years old). (**a**) For gross N mineralization, the 10 years old regrowth forest was significantly higher than the 40 years old forest (One way ANOVA with Tukey’s post hoc test *P* < 0.05) and slightly higher than the 20 years old forest (One way ANOVA with Tukey’s post hoc test *P* = 0.055). F value was 1.327 with degree of freedom of 3. (**b**) For gross nitrification, the pristine forest was significantly higher than all three regrowth forests (One way ANOVA with Tukey’s post hoc test *P* < 0.05). F value was 1.629 with degree of freedom of 3.

Changes in the internal soil N cycle as consequence of reforestation reflect alterations in the microbial and plant community during regrowth stage^21^. Rates of gross mineralization in the pristine forest at Cuniã (7.8 ± 4.7 µg N g^−1^ d^−1^; Fig. 1) are within the range of gross mineralization reported in other pristine tropical forests^22,23^. In a study in Eastern Amazon forest^24^ during the dry season, gross mineralization was measured *in situ* with a rate of 13.9 ± 3.8 and 7.2 ± 1.8 µg N g^−1^ d^−1^ from clay and sandy soils^24^ respectively, similar to the gross rate in the pristine forests in our study.

Along the chronosequence of forest regrowth gross N mineralization was nearly doubled in the youngest forest (10 years old; 14.8 ± 6.5 µg N g^−1^ d^−1^) but only half in the older regrowth forests (3.8 ± 2.1 µg N g^−1^ d^−1^) compared to the pristine forest (Fig. 1). A similar pattern was also observed in subtropical Australia, where gross N mineralization was 2 to 3 times higher in early monospecific forest plantation (5 years) than pristine forest and older (53 years) plantation^25^. In general, the main pattern seen in early successional forests is high rate of NH_4_^+^ release through mineralization^25,26^, although fewer contrasting results have also been reported^27–29^.

The observed change in gross N mineralization is not caused by SOM content. Across the chronosequence, the SOM content in pristine forest was significantly higher (P < 0.05) than in 10 years old regrowth, but not different from 20 and 40 years old regrowth forest (Table 1). This pattern is exactly the opposite as found for gross mineralization, hence the lowest SOM content was found in the forest with the highest gross N mineralization rate (10 years old regrowth forest). Instead, the quality of SOM^30,31^ might be more important for controlling N mineralization^24,32,33^. The C: N ratio, an indicator of the SOM quality and its degradation rate^34^, confirmed that, since the 10 years old regrowth had the lowest C: N ratio of 17.7 of the investigated forest soils (Table 1). Gross mineralization in early regrowth forests can be high due to the recent disturbance that redistribute SOM stored in deeper soil layers to soil surface^26^. Furthermore, the previous management, as slash-and-burn, degraded the SOM, releasing labile compounds that are easier to mineralize^21,35^. Subsequently, gross mineralization decreases over time, possibly due to depleting in labile SOM and are lower than in pristine forests due to reduced root exudation and rhizosphere priming^36–38^. After a fire event, soil texture might change, usually showing a decrease of clay and increase of sand content^39^. In addition, clay aggregates can change in terms of size and distribution in the soil. We only measured soil texture in the pristine forest, which had low clay content (4.9 ± 0.7%). Therefore, we expect small changes in soil texture along the chronosequence caused by the slash-and-burn practice, consequently hence having minimal effects on the measured microbial processes rates.

**Table 1:** Soil properties (mean ± SE) of pristine forest and three regrowth forests (10, 20 and 40 years old) at/near the Ecological Station of Cuniã, Rondônia (Brazil).

	Pristine	10 yrs.	20 yrs.	40 yrs.	F, degrees of freedom
pH	3.7 ± 0.04^a^ N = 14	3.4 ± 0.1^b^ N = 6	3.9 ± 0.04^c^ N = 6	3.8 ± 0.05^a,c^ N = 6	12.82, 3
GWC (%)	35.1 ± 0.8^a^ N = 51	22.0 ± 0.9^b^ N = 17	35.8 ± 1.5^a^ N = 15	30.8 ± 1.2^a^ N = 14	Non-parametric data
SOM (%)	7.5 ± 0.4^a^ N = 50	5.1 ± 0.7^b^ N = 11	8.0 ± 1.8^a,b^ N = 10	6.8 ± 1.2^a,b^ N = 11	6.676, 3
TC (%)	4.4 ± 0.3^a^ N = 51	2.9 ± 0.4^b^ N = 12	4.7 ± 1.^a,b^ N = 10	3.9 ± 0.7^a,b^ N = 11	4.074, 3
TN (%)	0.19 ± 0.01^a^ N = 51	0.17 ± 0.02^a^ N = 12	0.20 ± 0.01^a^ N = 12	0.17 ± 0.02^a^ N = 11	0.7086, 3
C: N	24., 8 ± 1, 6^a^ N = 51	17., 7 ± 2, 1^a^ N = 12	28. ± 3.7^a^ N = 9	25.0 ± 4.8^a^ N = 11	Non-parametric data

The letters a, b and c represent the values that are statistically significantly different in the four studied sites One way ANOVA with Tukey’s post hoc test (P < 0.05) was used for parametric soil properties (pH, SOM, TC, TN) and Kruskal-Wallis test with Dunn’s post hoc test, *P* < 0.05 for non-parametric (GWC and C: N). The F values and degrees of freedom were provide for parametric data.

Our results indicate that plant available N is sustained during forest regrowth due to enhanced gross N mineralization. High N mineralization in the early successional stage provides plant available N, overcoming a potential N limitation of forest regrowth. With time, N demand will decrease, which is also reflected in the decrease in gross N mineralization over time of forest regrowth found by us (Fig. 1) and others^25^.

### Conservative N cycling in tropical regrowth forests through decreased nitrification

**Figure 2:**
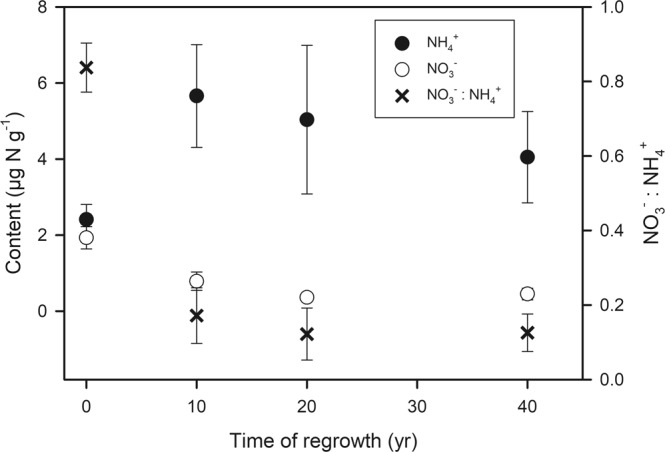
Content of soil NH_4_^+^ and NO_3_^−^ as well as NO_3_^−^: NH_4_^+^ ratio in pristine forest (set to t = 0 years) and three regrowth forests (10, 20 and 40 years) at the Ecological Station of Cuniã, Rondônia (Brazil). The contents were calculated from the first extraction after ^15^N labelling by subtracting the amount of tracer recovered (based on ^15^N enrichment). The black circle represents NH_4_^+^ content, the empty circle represents NO_3_^−^ content and the symbol X represents the NO_3_^−^: NH_4_^+^ ratio. The unit of the N contents is µg N g^−1^ SDW and the values represent mean ± standard error.

Secondary forests exhibit a more conservative N cycle compared to pristine forests in the Amazon region, indicated by the shift in the dominant inorganic N form in the soil towards NO_3_^−^ (refs^8,40^), which is also observed at the chronosequence at Cuniã (Fig. 2). We show here that the underlying process is a change in gross nitrification, which was significantly lower in all stages of forest regrowth than the pristine forest in our chronosequence (Fig. 1). Gross nitrification rates of 3.27 ± 1.14 µg N g^−1^ d^−1^ in the pristine forest at Cuniã are in accordance with rates reported in earlier studies, which reported *in situ* gross nitrification in the range of 0.5 to 5.2 µg N g^−1^ d^−1^ (e.g. refs^22,24,41^).

Gross nitrification rates were lower in all regrowth stages, than in the pristine forest (Fig. 1), justifying the measured low soil NO_3_^−^ content (Fig. 2), which confirms the idea of N retention and conservation during ecosystems succession^8^. This could be related to the alteration of the soil microbial community. In the Amazon region a higher abundance of nitrifiers was found in a pristine forest in comparison to regrowth forest soils^42^, explaining the higher rates in pristine forest. In addition, an enhanced plant N demand, competing with the nitrifiers for NH_4_^+^, could also contribute to a low gross nitrification in regrowing forests.

**Figure 3:**
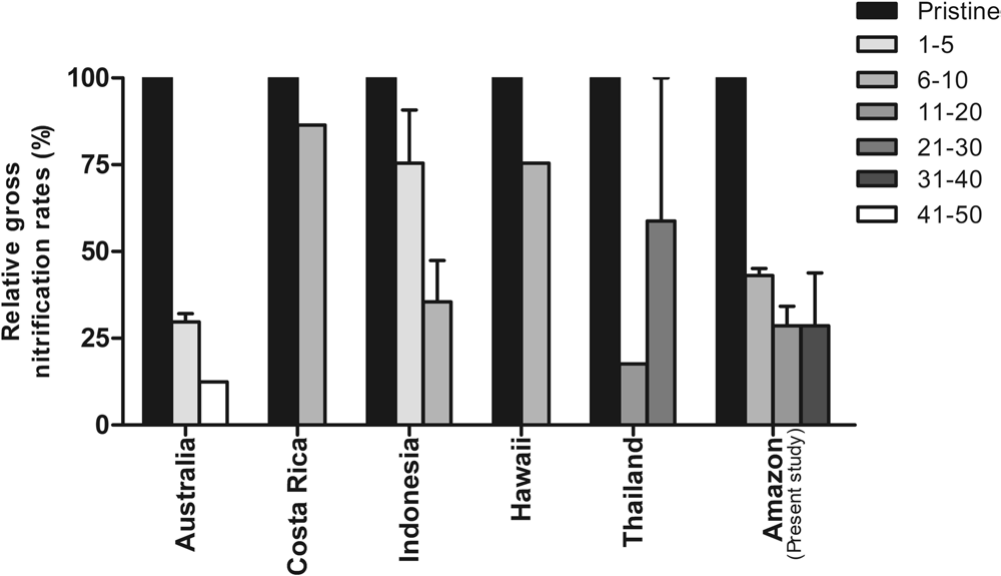
Relative gross nitrification rates in different tropical and subtropical pristine and secondary forests around the world. The figure compiles relative nitrification rates from pristine forest soil (black bar representing the highest nitrification rate in percentage) and secondary forests (plantation or regrowth) of different age (bars with different tones of gray). Data from refs^22,25,28,43,44^ and the present study.

The pattern of gross nitrification observed in this study, based on pseudo-replicated field experiment, is consistent and in agreement with studies from other chronosequences of tropical and sub-tropical forests^22,25,28,29,43,44^ (Fig. 3), which enables generalizations. Although the magnitude of gross nitrification varies between the different studies, consistently higher gross nitrification rate in pristine than secondary forests has been found, corroborating gross nitrification as the most suitable N process to evaluate recovery of tropical forest ecosystems.

### Nitrogen cycling along a tropical forest chronosequence

Tropical regrowing forests are characterized by a closed N cycle with low risk for N losses, indicated by decreased NO_3_^−^: NH_4_^+^ ratios^7,8^, which was also observed in this study (Fig. 2). Here, we provide a mechanistic understanding of the biogeochemical processes responsible for the evolution of the N cycle under forest regrowth. The consistent pattern reported for gross nitrification (Figs 1 and 3) explains the observed pattern of low NO_3_^−^: NH_4_^+^ ratios in regrowing forests. The relative excess in NO_3_^−^ in pristine forests promotes N losses by leaching and gaseous emission^23,24,45,46^. Regrowing forests, on the other hand, have a tighter N cycle with decreased N losses and enhanced N retention^47^.

Particularly the results from an investigation in a pristine sub-tropical forest and two forest plantations of different age in Australia are strikingly similar to our study^25^ (Fig. 3). Early regrowth forests are in both studies characterized by high rates of gross N mineralization and low rates of gross nitrification (Fig. 2), showing a decoupling of these two processes. As a consequence, inorganic N in the young forests is mainly in the form of NH_4_^+^, which leads to reduced N losses^24,48^, but maintains availability of N for plant uptake. Older regrowing forests, have a lower N demand^49^ and not only nitrification but also mineralization rates are low^25^ (Fig. 1).

**Figure 4:**
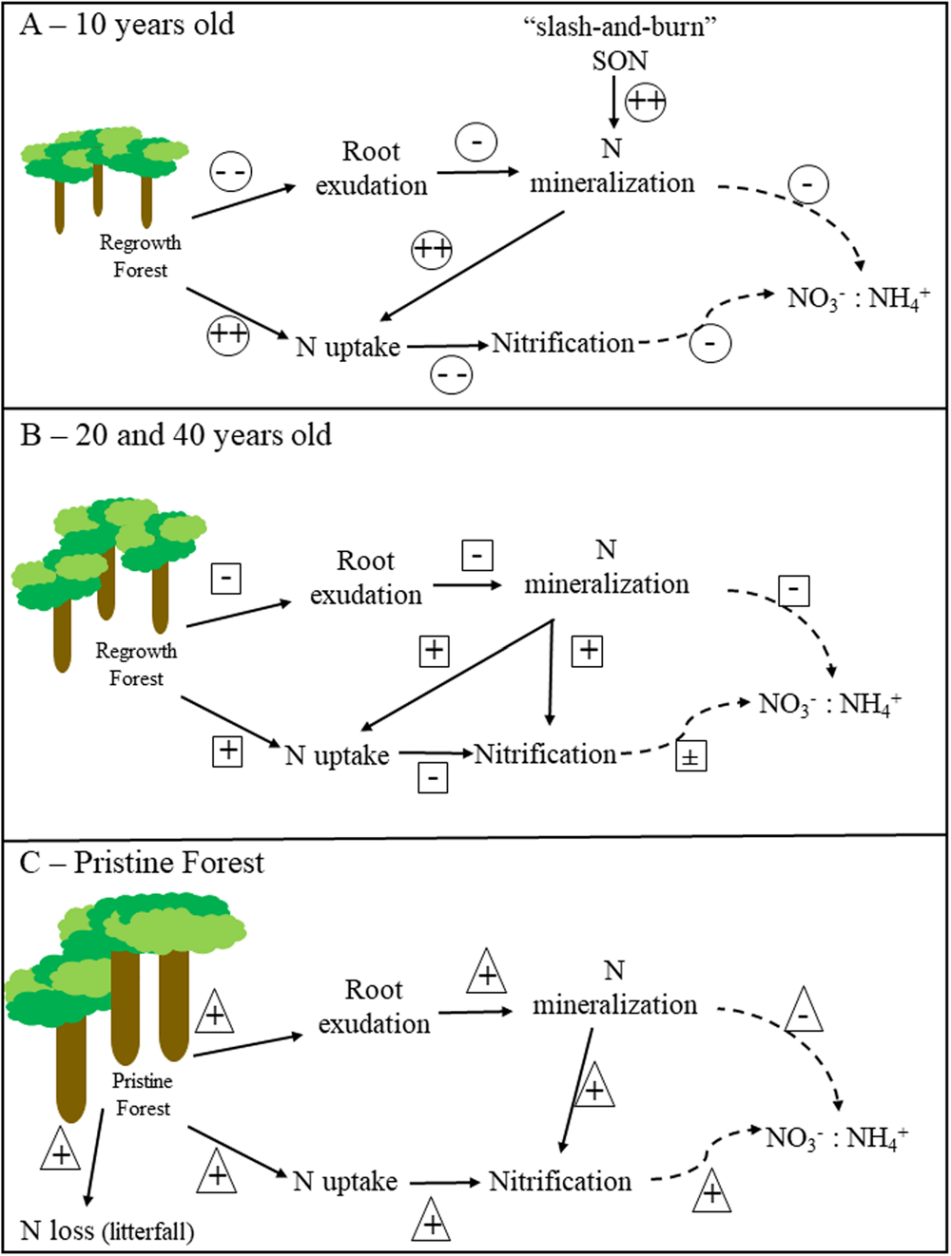
Conceptual model of N cycling along a forest chronosequence in Amazon region, (**a**,**b**). Nitrogen pathways in regrowing forest soils of different ages after one time disturbance by slash-and-burn. In the early regrowth forest (10 years; **a**) a new source of labile N from the burning of biomass stimulates gross mineralization and, as consequence of investment in forest growth, higher N uptake by plants. Nitrification and N uptake receiving support from N mineralization in 20 and 40 years old regrowth forests (**b**). In pristine forest (**c**) root exudations stimulates mineralization, which supports nitrification. See text for more details.

Although we did not directly investigate this, our results infer that plants are probably crucial in regulating the observed pattern of dominant N pathways during the forest regrowth in this part of the Amazon (Fig. 4). Root exudation and plant N uptake control the availability of inorganic N by affecting N cycling processes. The root exudation of labile organic compounds in the pristine forest provides not only a substrate for N mineralization but can stimulate gross mineralization further by rhizosphere priming^37,38,50^ (Fig. 4c). In regrowing forests with lower tree biomass, root exudation is lower, thereby reducing the effects on gross mineralization (Fig. 4a,b). In the youngest regrowth forest, this negative effect is though more than compensated for by the presence of labile SOM from the slash-and-burn practices^21,35^. The N assimilation in biomass is larger in regrowth in comparison to pristine forests, which have more N loss from litterfall than regrowth forests^40^. Because of that, the net uptake (gross N uptake minus N loss) is higher in regrowth (Fig. 4a,b) in comparison to pristine^51^ (Fig. 4c), decreasing the availability of NH_4_^+^ for nitrifiers, leading to a decrease in nitrification. As a consequence, the NO_3_^−^: NH_4_^+^ ratio will vary according to the forest status.

### Relevance of gross nitrification as an indicator of forest recovery

Gross N mineralization and gross nitrification are sensitive to environmental changes and ecosystem disturbance. Our findings suggest that 20 years after slash-and-burn disturbance gross N mineralization process decreases to closer rates to pristine stage. On the other hand, gross nitrification did not recovery even after 40 years (Fig. 1). This result indicates that the time for gross nitrification to recover to pristine conditions is much longer, suggesting that this process is more sensitive to disturbances. As a consequence, N is retained in the soil as NH_4_^+^, a plant available form, which is less prone to leaching processes. It is important to highlight the small range of environmental conditions evaluated here, such as season of the year associated to the precipitation variability, limited spatial replication, and absence of some characteristics of soil (i.e. soil texture), which can be a source of variation and spatial limitations. In the Eastern Amazon, Sotta *et al*.^24^ did not find differences in gross soil N cycling between clay and sand soil, neither between seasons. Moreover, the environmental factors probably have stronger influence on the magnitude than the patterns of the N cycle rates. Our findings have shown similar gross nitrification patterns as other tropical chronosequence forests (Fig. 3), which gives confidence in the robustness of the observed pattern. These findings combined demonstrate the sensibility of nitrification to disturbances in different tropical forests around the world and highlights the importance of gross nitrification as being the best mechanism to evaluate the evolution and recovery of N cycling in soils of secondary succession forests.

## Methods

### Study area

The study was carried out at and near the Ecological Station of Cuniã, Porto Velho municipality, Rondônia, Brazil (08°06′23″ S and 63°28′59″ W). The station was established for conservation and scientific research in 2001 in one of the Brazilian states with highest deforestation rate in the Amazonian region between 1980 and 1990^52^. The area of the station corresponds to 125,849.23 hectares of open rainforest dominated by hardwood with abundance of palms^20^. The soils studied were classified as Plinthosols, iron-rich and humus-poor and predominance of kaolinitic clay^19^. Inside the station, there is an area of 2500 ha previously defined for sampling and used in long-term monitoring. The mean annual precipitation in this region is 2500 mm, the rain season occurs from October to April, and the dry season from June to August. The mean annual temperature is around 26 °C^53^.

To investigate the *in situ* gross N transformations in intact soils of pristine and regrowth forest, one pristine forest and three regrowth forests with an age of 10, 20 and 40 years after slash-and-burn practice were chosen. The pristine forest was inside a grid of 1 km^2^ and was within the long-term monitoring site, as was the 40 years old regrowth forest (3–4 km from the pristine plot). The other two regrowth forests (approximately 10 and 20 years old; *personal communication* by local farmer) were located in the surrounding area, 10–12 km away.

### *In situ*^15^N labelling

To investigate the *in situ* gross N transformations in intact soil, with an intact rhizosphere, a ^15^N labelling using the “virtual soil core” approach^18^ was conducted at the beginning of the dry season in April 2013. Earlier studies on tropical forest soils^24,41^ found no differences in gross N rates between dry and wet season.

In the pristine forest seven plots in two straight lines, 1 km apart, were established with 10 m distance between plots. In the regrowing forests, three plots were randomly chosen with a distance of 10 m either in a straight line (40 years old) or in a triangle (10 and 20 years old), which was mainly governed by accessibility. Each plot was a pseudoreplication and, in each of them, two sets of a paired labelling spots were establish receiving a solution containing NH_4_^+^ and NO_3_^−^ with one of the N species enriched with ^15^N at 99% (Supplementary Fig. 1a).

Each spot received eleven 1 mL injections of ^15^N solution in a circular area of 7 cm in diameter, homogenously distributed into the soil underneath the litter to a depth of 9 cm using a 1 mL syringe and 9 cm spinal needle^18^ (Supplementary Fig. 1b). The total amount added corresponded to 1.73 µg NH_4_^+^-N and 0.86 µg NO_3_^−^-N per gram dry soil. One of the paired labelling spots was sampled immediately after labelling (t0) and the second one 24 hours (t24) after labelling. Soil sampling was conducted in the inner 4 cm labeling spot. The larger labeling area provides a buffer zone around the sampling^18^.

The intact soil samples were immediately transported to the field laboratory, where they were gently broken by hand to remove stones, leaves and large roots by tweezers. After sieving, 50 grams of each soil sample was added to a brown plastic bottle together with 100 mL of 1 M KCl, placed on a shaker for 1 hour, and lastly filtered through MN 615 filter paper (Macherey-Nagel).

The remaining soil was dried later in the laboratory to measured physicochemical soil properties; gravimetric water content (GWC) by drying at 100 °C, the soil organic matter content (SOM) by loss-on-ignition, and the total C and N (TC and TN) was measured on an elemental analyser coupled to an Isotope Ratio Mass Spectrometer (IRMS) (20–22, Sercon Ltd., Cheshire, UK). The pH was measured in the 1 M KCl extracts with a pH meter (691, Metrohm AG, Herisau, CH). Concentrations of NH_4_^+^ and NO_3_^−^ in KCl extracts were measured on flow injection analyser (FIAstar 5000, Foss Tecator AB, Brazil). The soil texture was determined in the pristine soil using a laser type granulometer (Malvern Mastersizer 2000, Malvern Instruments SA, Orsay cedex, France). The soil properties are showed in Table 1.

For analysis of ^15^N abundance, NO_3_^−^ in extracts was measured using the automatic measuring method Sample Preparation of Inorganic N compounds Mass Spectrometry (SPINMAS)^54^ at UFZ Halle. The ^15^N abundance of NH_4_^+^ was analysed using the micro-diffusion technique^55^, in which NH_4_^+^ is trapped in acidified glass fibre filters and analysed using an elemental analyser (ANCA-GSL, PDZ Europa, UK) coupled to the same IRMS as above, conducted at the Stable Isotope Facility at the University of California, Davis.

### Data analysis

Gross N mineralization and nitrification rates were calculated for each plot using the analytical ^15^N tracing model^56^, using data from the ^15^NH_4_^+^ labelling for gross mineralization and ^15^NO_3_^−^ labeling for gross nitrification:

1 $$N\,{\rm{transformation}}\,{\rm{rate}}=\frac{{N}_{0}-{N}_{t}}{t}\times \frac{{\mathrm{log}}_{10}(\frac{a{^{\prime} }_{0}}{a{^{\prime} }_{t}})}{{\mathrm{log}}_{10}({N}_{0}/{N}_{t})},$$

where *N*_0_ and *N*_*t*_ are soil NH_4_^+^ or NO_3_^−^ content at time zero and *t*, respectively, t is the time in days. The *a*′_0_ and *a*′_*t*_ are the excess ^15^N fractions of NH_4_^+^ or NO_3_^−^ at time zero and *t*, respectively. All raw data used in the equation 1 to calculate gross rates are presented Supplementary Table 1. Average gross rates were calculated per forest type and are presented on soil dry weight (SDW). A one-way analysis of variance (ANOVA) with Tukey’s post-test (P < 0.05) was carried out to examine the differences between the four forest sites.

The Normality test (Shapiro-Wilk) was used to examine the normality of soil properties. As some of our data, such as GWC, TN, soil NH_4_^+^ and NO_3_^−^ content were not normally distributed, the Kruskal-Wallis test with Dunn’s post-test (P < 0.05) was conducted to examine the difference between the four forest sites. Data of pH, SOM and TC showed a normal distribution and one-way analysis of variance (ANOVA) was conducted. All the analyses were conducted using GraphPad Prism (Version 5.01, GraphPad Software, Inc.).

## Supplementary information

Supplementary information of Evolution of nitrogen cycling in regrowing Amazonian rainforest
