# Supplementary material for: Evolution of nitrogen cycling in regrowing Amazonian rainforest
Source: Sci Rep. 2019 Jun 12;9:8538. doi: 10.1038/s41598-019-43963-4 (PMC6561906; doi:10.1038/s41598-019-43963-4)
Supplement: Supplementary file 1 — Supplementary information of Evolution of nitrogen cycling in regrowing Amazonian rainforest [file 41598_2019_43963_MOESM1_ESM.pdf]

Supplementary information

Evolution of nitrogen cycling in regrowing Amazonian rainforest

Viviane Figueiredo, Alex Enrich-Prast, Tobias Rütting

Content:

SI-1 – Figure S1: Soil labelling design.

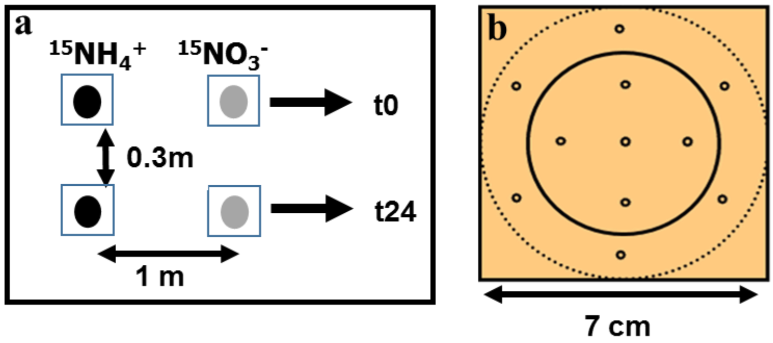

Figure S1 I Scheme of soil labelling with ^{15}N forms. a) The two ^{15}N solutions ($^{15}NH_4^+$ and $^{15}NO_3^-$ enriched) are applied in two different transects 1 m apart from each other. The solutions are added in the soil *in situ*, and each transect has two labelled spots, which was destructively samples immediately after labelling (t0) and after 24 hours (t24). **b)** Each labelled spot received the ^{15}N solution distributed in 11 injections of 1 mL each. The black circle represents the soil area that is taken out after the labelling, and the dotted line represents the buffer area, to guarantee a homogeneous labelling.

Supplementary Table

Code ($^{15}NH_4$ added)	$^{14}NH_4$ (µg/g)	$^{15}NH_4$ excess (%)	Code ($^{15}NO_3$ added)	$^{14}NO_3$ (µg/g)	$^{15}NO_3$ excess (%)
P.a.A.0	4.724	14.72	P.a.N.0	2.612	23.53
P.a.A.1	1.550	1.72	P.a.N.1	4.277	10.18
P.b.A.0	2.693	36.27	P.b.N.0	2.198	24.65
P.b.A.1	0.701	3.28	P.b.N.1	4.689	11.33
P.c.A.0	4.765	19.53	P.c.N.0	4.509	19.19
P.c.A.1	0.563	2.22	P.c.N.1	8.572	9.39

P.d.A.0	2.208	12.40	P.d.N.0	2.659	35.78
P.d.A.1	3.803	5.31	P.d.N.1	4.395	0.70
P.e.A.0	2.626	43.99	P.e.N.0	2.161	36.02
P.e.A.1	4.560	2.48	P.e.N.1	3.053	12.69
P.f.A.0	2.791	35.02	P.f.N.0	2.401	30.52
P.f.A.1	2.066	1.22	P.f.N.1	4.706	9.24
P.g.A.0	3.358	36.17	P.g.N.0	1.970	23.14
P.g.A.1	11.707	0.65	P.g.N.1	3.408	12.30
R10.a.A.0	9.288	21.51	R10.a.N.0	1.067	69.57
R10.a.A.1	6.872	1.84	R10.a.N.1	1.572	23.67
R10.b.A.0	7.420	8.70	R10.b.N.0	1.805	36.59
R10.b.A.1	2.585	1.11	R10.b.N.1	1.453	17.51
R10.c.A.0	3.628	17.74	R10.c.N.0	1.495	39.59
R10.c.A.1	6.944	2.81	R10.c.N.1	1.980	17.82
R20.a.A.0	9.749	10.98	R20.a.N.0	0.527	62.02
R20.a.A.1	3.316	3.78	R20.a.N.1	0.265	3.39
R20.b.A.0	4.950	8.17	R20.b.N.0	0.569	30.94
R20.b.A.1	2.042	4.65	R20.b.N.1	0.380	9.91
R20.c.A.0	2.166	10.38	R20.c.N.0	0.974	48.64
R20.c.A.1	2.296	2.91	R20.c.N.1	0.747	15.63
R40.a.A.0	7.258	13.10	R40.a.N.0	1.365	48.41
R40.a.A.1	3.336	6.98	R40.a.N.1	3.259	22.03
R40.b.A.0	2.964	26.37	R40.b.N.0	1.223	60.77
R40.b.A.1	4.713	5.88	R40.b.N.1	1.840	34.30
R40.c.A.0	4.697	21.01	R40.c.N.0	0.868	79.69
R40.c.A.1	2.952	12.64	R40.c.N.1	0.715	72.37

Table S1. Concentration of $^{14}NH_4$ and $^{14}NO_3$ (μg/g) and excess ^{15}N fractions of $^{15}NH_4$ and $^{15}NO_3$ (%) in the soil from each virtual core evaluated. P, R10, R20 and R40 represent soils from pristine forest, 10, 20 and 40 years old regrowth forests, respectively; a-g represent the transects; A and N represent the soil which was labelled with ^{15}Ammonium or ^{15}Nitrate; 0 and 1 represent the corresponding time of each concentration after the soil had been labelled. The values presented in this table were used to calculate the gross rates of N mineralization and nitrification with equation 1.

Table S2. Initial ^{15}N recovery:

Forest age	NH_4^+ (%)	NO_3^- (%)
Pristine	51	81
10 yrs.	62	76
20 yrs.	32	37
40 yrs.	51	80
